# Functional organization of mouse primary auditory cortex in adult C57BL/6 and F1 (CBAxC57) mice

**DOI:** 10.1038/s41598-020-67819-4

**Published:** 2020-07-02

**Authors:** Zac Bowen, Daniel E. Winkowski, Patrick O. Kanold

**Affiliations:** Department of Biology, University of Maryland, 1116 Biosciences Res. Bldg., College Park, MD 20742 USA

**Keywords:** Cortex, Sensory processing

## Abstract

The primary auditory cortex (A1) plays a key role for sound perception since it represents one of the first cortical processing stations for sounds. Recent studies have shown that on the cellular level the frequency organization of A1 is more heterogeneous than previously appreciated. However, many of these studies were performed in mice on the C57BL/6 background which develop high frequency hearing loss with age making them a less optimal choice for auditory research. In contrast, mice on the CBA background retain better hearing sensitivity in old age. Since potential strain differences could exist in A1 organization between strains, we performed comparative analysis of neuronal populations in A1 of adult (~ 10 weeks) C57BL/6 mice and F1 (CBAxC57) mice. We used in vivo 2-photon imaging of pyramidal neurons in cortical layers L4 and L2/3 of awake mouse primary auditory cortex (A1) to characterize the populations of neurons that were active to tonal stimuli. Pure tones recruited neurons of widely ranging frequency preference in both layers and strains with neurons in F1 (CBAxC57) mice exhibiting a wider range of frequency preference particularly to higher frequencies. Frequency selectivity was slightly higher in C57BL/6 mice while neurons in F1 (CBAxC57) mice showed a greater sound-level sensitivity. The spatial heterogeneity of frequency preference was present in both strains with F1 (CBAxC57) mice exhibiting higher tuning diversity across all measured length scales. Our results demonstrate that the tone evoked responses and frequency representation in A1 of adult C57BL/6 and F1 (CBAxC57) mice are largely similar.

## Introduction

The cerebral cortex is uniquely adapted to encode behaviorally relevant stimuli and generate appropriate behavioral actions. The primary auditory cortex (A1) plays a key role for sound perception since it represents one of the first cortical processing stations for sounds. Recent studies have investigated the functional organization and circuitry of mouse auditory cortex^1–6^. A classic hallmark of A1 organization in all species has been the discovery of tonotopic maps which describe a smooth distribution of tone preference across the surface of the primary and secondary auditory fields when probed with low spatial resolution techniques^6–18^. However, the application of large-scale high-resolution optical techniques in mice or rats using synthetic dyes (OGB1, Fluo4, or Cal-520) or sensitive genetically encoded Ca^2+^ indicators (GCaMP6) has shown that on the cellular level the organization of tuning in A1 is more heterogeneous than previously appreciated^1–6, 19–22^ suggesting that at least in rodent layer 2/3 (L2/3) functional tonotopic maps might be fractured. The precise degree of heterogeneity varies slightly between these studies, and this difference might be due to differences in anesthetic condition, cell inclusion criteria, tonal stimuli used, and/or species.

Many functional studies in mouse have been performed on mice on the C57BL/6 background. However, the C57BL/6 strain carries a mutant *cdh23* allele^23–29^ which leads to a progressive loss of hair cells from the basal turn of the cochlea ^30^, making these mice an ideal model of presbycusis^31–35^. In contrast, mice of the CBA strain or F1 offspring of C57BL/6 mice and CBA mice retain normal hearing into adulthood^31–36^. Thus, potential hearing loss present at the ages studied in C57BL/6 mice could have contributed to the observation of local frequency preference heterogeneity. We aimed to test if the laminar differences in functional responses and organization present under anesthetized conditions in C57BL/6 mice were also present in awake animals and if such organizational features were also present in F1 (CBAxC57) mice.

We performed in vivo 2-photon imaging experiments in awake mice of C57BL/6 strain as well as CBA background (CBAxC57 F1) and quantified the functional responses across L2/3 and L4 of A1. We focused on young adult animals (~ 2–3 months old) which is the age frequently used in functional studies, especially those involving mouse behavior^1–6,37–41^. To compare strains we imaged the mid-frequency region of A1 (4–16 kHz) which is also where mice are most sensitive to sound^42^. We find that A1 of F1 (CBAxC57) mice contains more neurons tuned to high frequencies and a higher number of significant tone-responses overall, including a higher proportion of off-responses^6^. Moreover, on the single cell level, bandwidth was lower in C57BL/6 mice while sound pressure level dependent analysis revealed an increased gain in F1 (CBAxC57) mice. On the population level, we find that in both strains the local heterogeneity is higher in L2/3 than in L4 but that heterogeneity is slightly higher in F1 (CBAxC57) mice than C57BL/6 mice. Our observations show that while there are differences in the tuning of single neurons between mice, there remains laminar differences in both frequency selectivity and local heterogeneity of frequency preference present in both strains. Our results here along with previously published results show that the local heterogeneity of frequency preference is present in both anesthetized and awake mice and across mouse strains. Thus, the heterogeneity of pure tone frequency selectivity forms an organizational principle of auditory cortex organization in mice.

## Methods

All animal procedures and experiments were approved by the University of Maryland Institutional Animal Care and Use Committee to be in accordance with applicable guidelines and regulations.

Mice were housed under a reversed 12 h-light/12 h-dark light cycle with ad libitum access to food and water. Imaging experiments were generally performed near the end of the light and beginning of the dark cycle. We imaged adult mice (C57BL/6: P71-P93; F1 (CBAxC57): P66-P93 and P166-P178 at time of imaging). We specifically included older F1 (CBAxC57) mice (P166-P178) to ensure that the peripheral hearing loss experienced by C57BL/6 mice never became evident in F1 (CBAxC57) mice. Mice of both sexes were used based on their availability not by any biased selection.

### Animals

Mice used for this study expressed the genetically encoded calcium indicator GCaMP6s^49^. We used transgenic mice that expressed GCaMP6s either under the Thy1 promoter (Jax: 024275, GP4.3) or a transgenic mouse line that conditionally expresses GCaMP6s when crossed to a mouse driver line expressing Cre recombinase under the control of Emx1 (GCaMP6s mouse: Jax: 024115; Emx1-Cre mouse: Jax: 005628). Mice on this background do not show epileptiform activity in A1^43^. All analyses were initially performed on Emx-TetO-GC6 and Thy1-GC6 separately for the C57BL/6 mice. Results from the two GCaMP6s lines were pooled when in agreement and were displayed separately in any results where they differed. Mice on the C57BL/6 background show early onset age related hearing loss^31–36^. To compare these mice with mice that do not develop early hearing loss, we used the F1 generation of a hybrid mouse line CBA/CaJ (Jax: 000654) crossed with C57BL/6-Thy1 which have normal hearing^36^. During imaging, awake mice were head-fixed and restricted in a tube. L2/3 and L4 datasets were always obtained sequentially in the same mouse, meaning the experiment (sound presentations) was conducted in one layer and then sequentially conducted in the other layer. The two transgenic lines exhibited robust indicator expression in both L2/3 and L4^43^.

### Surgery and animal preparation

Surgical procedures were performed as previously reported^43^. Mice were given a subcutaneous injection of dexamethasone (5 mg/kg) at least 2 h prior to surgery to reduce potential inflammation and edema from surgery. Mice were deeply anesthetized using isoflurane (5% induction, 2% for maintenance) and given subcutaneous injections of atropine (0.2 mg/kg) and cefazolin (500 mg/kg). Internal body temperature was maintained at 37.5 °C using a feedback-controlled heating blanket. The scalp fur was trimmed using scissors and any remaining fur was removed using Nair. The scalp was disinfected with alternating swabs of 70% ethanol and betadine. A patch of skin was removed, the underlying bone was cleared of connective tissue using a bone curette, the temporal muscle was detached from the skull and pushed aside, and the skull was thoroughly cleaned and dried. A thin layer of cyanoacrylate glue (VetBond) adhesive was applied to the exposed skull surface and a custom machined titanium head plate (based on the design described in Guo et al.^44^ was affixed to the skull overlying the auditory cortex using VetBond followed by dental acrylic (C&B Metabond). A circular craniotomy (~ 3 mm diameter) was made in the center opening of the head plate and the patch of bone was removed. Then, a chronic imaging window was implanted. The window consisted of a stack of 2–3 mm diameter coverslips glued with optical adhesive (Norland 71, Edmund Optics) to a 5 mm diameter coverslip. The edges of the window between the glass and the skull were sealed with a silicone elastomer (Kwik-Sil) and then covered with dental acrylic. The entire implant except for the imaging window was then coated with black dental cement created by mixing standard white powder (Dentsply) with iron oxide powder (AlphaChemical, 3:1 ratio)^45^. Meloxicam (0.5 mg/kg) and a supplemental dose of dexamethasone were provided subcutaneously as a post-operative analgesic. Animals were allowed to recover for at least 1 week prior to the beginning of experiments.

### Acoustic stimulation

Sound stimuli were produced and presented as described previously^43^. Sounds were synthesized in MATLAB using custom software (courtesy of P. Watkins, UMD), passed through a multifunction processor (RX6, TDT), attenuated (PA5, Programmable Attenuator), and delivered via ES1 speaker placed ~ 5 cm directly in front of the mouse. The sound system was calibrated between 2.5 and 80 kHz and showed a flat (± 3 dB) spectrum over this range. Overall sound pressure level (SPL) at 0 dB attenuation was ∼90 dB SPL (for tones). Sounds were played at four sound levels (20, 40, 60, and 80 dB SPL). Auditory stimuli consisted of sinusoidal amplitude-modulated (SAM) tones (20 Hz modulation, cosine phase), ranging from 3 to 48 kHz. For widefield imaging, the frequency resolution of the stimuli was 1 tone/octave; for 2-photon imaging, the frequency resolution was 2 tones/octave (0.5 octave spacing). Each of these tonal stimuli was repeated five times with a 4–6 s interstimulus interval for a total of either 75 (widefield) or 135 (2-photon) iterations.

### Widefield and 2-photon imaging

Widefield imaging was used to identify the large scale tonotopic organization as previously^5,6,43^. To study cellular neuronal activity using 2-photon imaging, we used a scanning microscope (Bergamo II series, B248, Thorlabs) coupled to a pulsed femtosecond Ti:Sapphire 2-photon laser with dispersion compensation (Vision S, Coherent). The microscope was controlled by ThorImageLS software. The laser was tuned to a wavelength of λ = 940 nm in order to excite GCaMP6s. Green signal was collected through a 16 × 0.8 NA microscope objective (Nikon). Emitted photons were directed through a 525/50–25 (green) band pass filter onto GaAsP photomultiplier tubes. The field of view was 370 × 370 μm^2^. Imaging frames of 512 × 512 pixels (0.52 μm^2^ pixel size) were acquired at 30 Hz by bi-directional scanning of an 8 kHz resonant scanner. Beam turnarounds at the edges of the image were blanked with a Pockels cell (Conoptics). The average power for imaging in both L2/3 and L4 was <  ~ 70 mW, measured at the sample plane. We imaged in the mid-frequency regions of A1 and validated this by measuring the median BF of cells in the imaging field.

### Data analysis

#### Widefield imaging analysis

Widefield image sequences were analyzed the same as previously reported^43^ using custom routines written in MATLAB (Mathworks). Images were parsed into trial-based epochs in which each frame sequence represented a single trial consisting of the presentation of a single sound frequency-intensity combination. For each trial, response amplitude (ΔF/F_0_) as a function of time was determined for each pixel using the formula ((F – F_0_)/ F_0_) where F corresponds to the time varying fluorescence signal at a given pixel and F_0_ was estimated by averaging the fluorescence values over 4 frames (~ 1 s) prior to sound onset for a given trial and pixel. For construction of sound-evoked response maps, the amplitude of the ΔF/F_0_ pixel response during 1 s after stimulus onset (~ 4 frames) was averaged across time and repetitions yielding an average response magnitude that was assigned to each pixel. Responsive areas in the average response maps were defined on a pixel-by-pixel basis as pixels in which the average brightness of the pixel during the 1 s after stimulus onset exceeded 2 standard deviations of the pixel brightness during the 1 s before the stimulus across stimulus repetitions.

#### 2-photon imaging analysis

2-photon image sequences were preprocessed as described previously^43^. Image sequences were corrected for x–y drifts and movement artifacts using either the TurboReg in ImageJ^46,47^ or discrete Fourier transform registration^48^ implemented in MATLAB (Mathworks). Neurons were identified manually from the average image of the motion corrected sequence. Ring-like regions of interest (ROI) boundaries were drawn based on the method described in Chen et al.^49^. Overlapping ROI pixels (due to closely juxtaposed neurons) were excluded from analysis. For each labeled neuron, a raw fluorescence signal over time (F_soma_) was extracted from the ROI overlying the soma. Neuropil (NP) subtraction was performed on the raw fluorescence of all soma ROIs (F_soma_)^50^. In short, the neuropil ROI was drawn based on the outer boundary of the soma ROI and extended from 1 pixel beyond the soma ROI outer boundary to 15 μm excluding any pixels assigned to neighboring somata. Thus, the fluorescence (F) used for analysis was calculated as F = F_soma_ – (α × F_NP_), where we used α = 0.9 to reduce fluorescence contamination from the neuropil^50^. The mean fluorescence for each neuron was calculated across frames and converted to a relative fluorescence measure (ΔF/F_0_), where ΔF = (F – F_0_). F_0_ was estimated by using a sliding window that calculated the average fluorescence of points less than the 50th percentile during the previous 10-s window (300 frames). Neurons in which the ΔF/F_0_ signal was significantly modulated by sound presentation were defined by ANOVA (p < 0.01) across baseline (pre-stimulus) and all sound presentation periods. Single neuron receptive fields (RF) were determined as the average ΔF/F_0_ response to each frequency-intensity combination across 5 stimulus repetitions during the stimulus window (1 s). Best frequency (BF) for each neuron was determined as the frequency that produced the largest average dF/F response regardless of sound pressure level. The characteristic frequency (CF) is the highest average dF/F response at the lowest sound level for which the neuron had a significant response.

Receptive Field Sum (RFS) was calculated by first only keeping values of the RF for which the neuron had a significant response above baseline (setting non-significant elements to zero). This RF was then normalized and summed across all elements to arrive at the RFS (Fig. 2A). For a perfectly selective neuron that only responded significantly to one frequency/SPL combination, the RFS would equal 1. The closer the RFS was to 1, the more selective the neuron. A higher RFS indicates a neuron that was highly responsive across a large area of its receptive field. The theoretical maximum value would be a neuron responding significantly and equally to all frequency/SPL combinations giving a value of 36, however this was never observed in our data.

In order to test how receptive fields with sparse, yet high dF/F values affect the RFS, we performed the same analysis using a fully binarized RFS, where each significant response assigns a value of 1 rather than a value between 0 and 1 with normalization. In this binarized analysis, each significant response is treated equally. The results using this binarized RFS measure (Supplementary Figure S1) follow the same trends as our original RFS measure. Therefore, any instances of significantly skewed receptive fields from abnormally high dF/F are either not drastically affecting the RFS results or are not prominent in the data.

Signal correlations were obtained for each neuronal pair by calculating the correlation coefficient of their frequency response areas (FRAs) utilizing MATLAB’s “corrcoef” function, which performs the following:

$$\rho \left(A,B\right)= \frac{1}{N-1}\sum_{i=1}^{N}\left(\frac{{A}_{i}-{\mu }_{A}}{{\sigma }_{A}}\right)\left(\frac{{B}_{i}-{\mu }_{B}}{{\sigma }_{B}}\right)$$

where *A* and *B* represent two FRAs each with *N* elements and *µ* and *σ* represent the mean and standard deviation, respectively. Noise correlations were obtained for each cell pair by finding the covariance of trial-to-trial response fluctuations about the mean. This was done by calculating an FRA for each trial block and calculating its difference from the true FRA (mean across trials). The “corrcoef” function was used on pairs of trial block fluctuation FRAs. The correlation coefficient values from each trial block were averaged to arrive at one noise correlation value for each neuronal pair.

## Results

**Figure 1:**
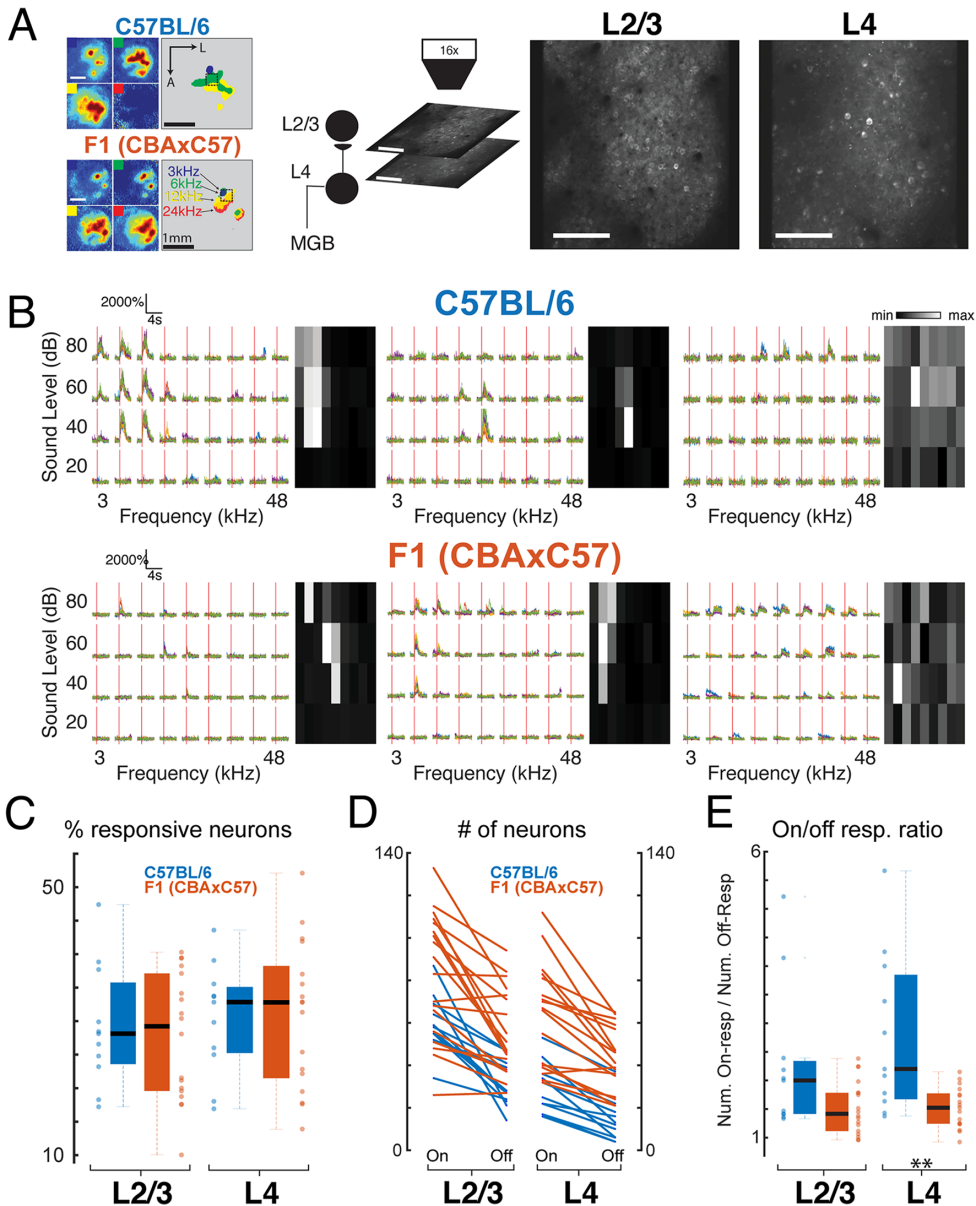
Neurons respond to a range of stimuli in both layers of both strains. **(A)** Left: Widefield imaging is used to identify mid-frequency regions. Panels show fluorescence to four different tones at 60 dB SPL and map on right shows overlay. Dashed boxes indicate mid-frequency region of A1. Right: Example 2-photon imaging FOVs in L2/3 and L4 of awake mouse auditory cortex. Scale bar 100 µm. **(B)** Example calcium traces and frequency response areas (Receptive Fields) for C57BL/6 mice (top) and F1 (CBAxC57) mice (bottom). Red vertical lines indicate tone-onset. Individual traces represent single trial calcium transients. **(C)** Percent of neurons in each FOV deemed significantly responding to at least one stimulus. Blue indicates C57BL/6 mice and orange indicates F1 (CBAxC57) mice. Mean and standard deviation for each distribution: C57_L2/3_ = 0.29 ± 0.09, F1_L2/3_ = 0.28 ± 0.09, p > 0.68 (two-sample *t*-test); C57_L4_ = 0.30 ± 0.08, F1_L4_ = 0.31 ± 0.11, p > 0.91 (two-sample *t*-test). **(D)** Number of neurons deemed On-responsive and Off-responsive in each FOV. Lines connect number of cells from the same FOV. **(E)** Ratio of on- to off-responsive neurons for each of the FOVs plotted in **D**. Median and IQR for L2/3 (non-normal): C57_L2/3_ = 2.00 median (0.92 IQR), F1_L2/3_ = 1.53 median (0.46 IQR), p > 0.05 (Wilcoxon rank sum). Mean and standard deviation for L4: C57_L4_ = 2.79 ± 1.39, F1_L4_ = 1.52 ± 0.35, p < 0.002 (two-sample *t*-test).

To investigate the functional responses of A1 neurons and their functional organization, we performed in vivo 2-photon imaging in awake animals chronically implanted with cranial windows^5,6^ using GCaMP6s expression. As done previously we performed widefield imaging to localize the imaging fields to A1 and identify mid-frequency areas^5,6^ (Fig. 1A, left) to guide field of view (FOV) location in 2-photon imaging experiments. We acquired 2-photon data sets in L2/3 and L4 sequentially in the same mouse ensuring that we imaged in the same tonotopic position in the mid-frequency region of both layers (Fig. 1A, right). We imaged 22 FOVs from C57BL/6 mice (11 L2/3, 11 L4) and 35 FOVs in F1 (CBAxC57) mice (18 L2/3, 17 L4). Imaging depths in C57BL/6 mice were 177 ± 17 µm (mean ± std) for L2/3 and 415 ± 7 µm for L4 and in CBA mice 160 ± 17 µm for L2/3 and 412 ± 5 µm for L4.

### A similar fraction of neurons responded to tonal stimuli across strains with respect to cortical layer

We first investigated the fraction of single neurons in L4 and L2/3 of A1 in mice of both strains that respond to tonal stimuli. We played a range of tones (3–48 kHz, half-octave spacing) at 4 sound pressure levels (80, 60, 40, and 20 dB) with five repetitions of each stimulus and found a wide variety of receptive fields, or frequency response areas (FRA), across the recorded neuronal populations (Fig. 1B). We identified tone-responsive neurons by a significant increase in the somatic fluorescent signal above baseline (dF/F) during stimulus presentation. We found that the proportion of neurons in each FOV with significant responses to at least one stimulus were similar (~ 25–35%) in both mouse strains and across cortical layers (Fig. 1C).

While many A1 neurons have a maximal response during the stimulus, A1 neurons can also have offset responses to tones (Fig. 1B, right) which can be separated from each other with long (> 1 s) stimuli^6^. We measured how many neurons had significant onset (tone-on) responses versus offset (tone-off) responses (Fig. 1D). We found that both F1 (CBAxC57) and C57BL/6 mice had tone-on as well as tone-off neurons with more tone-on neurons being present than tone-off neurons in both strains. Overall, L4 datasets had fewer lower number of neurons responding. We computed an on/off ratio as the ratio between the number of on-responsive neurons to the number of off-responsive neurons to quantify the disparity between on- and off-responsive neurons in each FOV. We found that the ratio is higher in both L2/3 and L4 of C57BL/6 mice than CBA mice (Fig. 1E) (p_L2/3_ < 0.06, Wilcoxon rank sum, p_L4_ < 0.002, two-sample t-test). Together, these results indicate that C57BL/6 mice have fewer off-responsive neurons relative to the number of on-responsive neurons, particularly in L4 than F1 (CBAxC57) mice. These results indicate that, in general, A1 of both strains contains a similar number of tonally responsive neurons but that L4 of F1 (CBAxC57) mice contains significantly more off-responsive neurons when compared to C57BL/6 mice.

### A1 neurons in F1 (CBAxC57) mice have a broader range of frequency preference than C57BL/6 mice

Having found that overall neuronal responsiveness was similar between strains, we next investigated the tuning properties of single neurons in both mouse strains. C57BL/6 mice show a progressive loss of high frequency hearing^30–35^. We thus investigated the possibility that A1 neurons in C57BL/6 mice showed altered frequency preference. Moreover, C57BL/6 mice show a loss of parvalbumin expression with age suggesting a decrease in inhibition ^51^. Since inhibition sharpens receptive fields, we also investigated the possibility that A1 neurons in C57BL/6 mice showed altered frequency selectivity.

**Figure 2:**
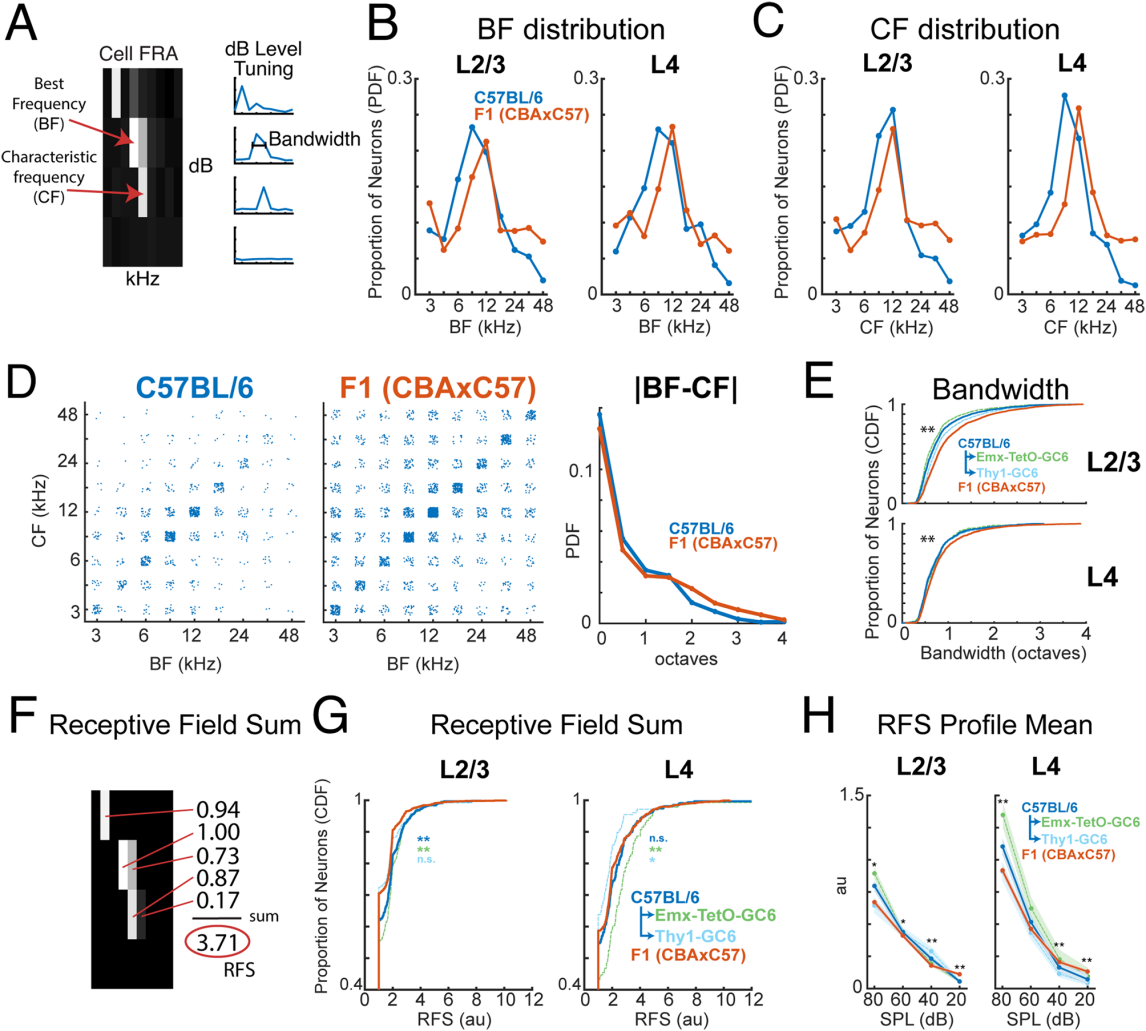
Larger bandwidth and more sensitive hearing in F1 (CBAxC57) mice. **(A)** Example FRA illustrating the measurement and computation of BF, CF, and bandwidth. **(B)** Histograms of BF distributions for C57BL/6 (blue) and F1 (CBAxC57) (orange) mouse strains in both L2/3 (left) and L4 (right). **(C)** Conventions as in **(B)**, but for CF. **(D)** BF plotted against CF for each neuron in both mouse strains. Each dot represents an individual neuron. |BF–CF| shows the absolute magnitude of difference between BF and CF for each mouse strain. **(E)** Cumulative distribution functions of bandwidth in C57BL/6 (blue) and F1 (CBAxC57) (orange) mouse strains in both L2/3 (left) and L4 (right). C57BL/6 breakdown of GCaMP6s lines also displayed for Emx-TetO-GC6 (green) and Thy-GC6 (light blue). ** Indicates significance between C57BL/6 and F1 (CBAxC57) at p < 0.01 (Wilcoxon rank sum test). **(F)** Example calculation of Receptive Field Sum. **(G)** Cumulative distribution functions of receptive field sum values in C57BL/6 (blue) and F1 (CBAxC57) (orange) mouse strains in both L2/3 (left) and L4 (right). Median and IQR for each distribution: C57_L2/3_ = 1.00 (0.83), F1_L2/3_ = 1.00 (0.66), C57_L4_ = 1.00 (1.18), F1_L4_ = 1.00 (0.92). **(H)** Receptive field sum split up by sound level. Sum is taken across rows in **(F)** and then averaged across all neurons. * and ** Indicates significance between C57BL/6 and F1 (CBAxC57) at p < 0.05 and p < 0.01 respectively (Wilcoxon rank sum test). Further statistical test details are included in Supplementary Table S1.

We first investigated the frequency preference of neurons by calculating the best frequency (BF) and characteristic frequency (CF) of each significantly responding neuron. The BF is the frequency that produced the highest response in each neuron regardless of sound pressure level, whereas the CF is the frequency that produced the highest response at the lowest sound pressure level that elicited a significant response (Fig. 2A).

We find that the BF and CF distributions are similar across L2/3 and L4 within each strain of mice (Fig. 2B, C). However, comparing strains showed that C57BL/6 mice had a somewhat broader distribution of represented BFs in the mid-frequency region (6–17 kHz) but a reduced number of neurons representing the high end of the frequency range (33–48 kHz) (Fig. 2B,C). These data indicate that the representation of tonal stimuli differs between mouse strains and that C57BL/6 mice show an under-representation of high-frequency stimuli consistent with high frequency hearing loss.

BF and CF measure different aspects of the FRA, and the BF can be affected by non-linear rate-level changes at the CF. If all single neurons have a linear increase in responses with increasing sound level, then CF and BF would be equal. Thus, we next probed the relationship between CF and BF for individual cells by plotting BF versus CF for each neuron in both mouse strains (combined across layers) (Fig. 2D). To account for differences in number of points, we also plot the probability density function of |BF–CF| across all neurons (Fig. 2D, right). We find that while CF and BF are mostly correlated in both mouse strains, C57BL/6 mice have fewer neurons with a mismatched BF and CF. This may be a consequence of C57BL/6 mice having worse detection of quieter sounds (lower SPL). As a result, each neuron may only have significant responses at one or two SPLs causing the maximal FRA response (BF) to occur at the lowest SPL with a significant response (CF). Moreover, the slightly broader BF vs CF distribution in F1 (CBAxC57) mice could also indicate that cells respond to a broad range of frequencies at varying SPLs, whereas cells in C57s mice are often sharply tuned.

We next assessed how frequency-selective A1 neurons were by calculating the bandwidth of the tuning curve at 70% of BF amplitude at the SPL for which BF was measured. We assessed frequency selectivity in C57BL/6 mice separately for two transgenic GCaMP6s lines, Thy1-GC6 and Emx1-TetO-GC6 (see “Methods”). Overall, C57BL/6 mice (both Thy1-GC6 and Emx1-TetO-GC6) had narrower bandwidths than the F1 (CBAxC57) mice in L2/3 and L4 (median and IQR: C57_L2/3_ = 0.64 (0.44), F1_L2/3_ = 0.79 (0.67), p < 2 × 10^–38^ (Wilcoxon rank sum); C57_L4_ = 0.61 (0.39), F1_L4_ = 0.68 (0.44), p < 6 × 10^–7^ (Wilcoxon rank sum). These results indicate that C57BL/6 mice had a higher selectivity for tonal stimuli (Fig. 2E).

A1 responses can have nonlinear changes in magnitude with increasing sound level, thus bandwidth measures might miss responses outside traditional “V-shaped” FRAs. Thus, to further investigate the frequency selectivity of each neuron, we developed a measure called the Receptive Field Sum (RFS) which is more inclusive when quantifying nonlinear tuning curves or receptive fields. For each neuron this measure is calculated by first only keeping values of the receptive field (RF) for which the neuron had a significant response above baseline (setting non-significant elements to zero). The remaining RF elements are then normalized and summed to arrive at the RFS (Fig. 2F). A lower RFS indicates that a neuron was more specific in its stimulus responsiveness. We find that in L2/3 neurons, F1 (CBAxC57) mice have a lower distribution of RFS values than C57BL/6 mice (p < 2.6 × 10^–5^, Wilcoxon rank sum) (Fig. 2G, left). When C57BL/6 mice are split up by GCaMP6s line, we find that Emx1-Cre based mice have lower RFS values than F1 (CBAxC57) (p < 2.7 × 10^–9^, Wilcoxon rank sum), yet Thy1-GC6 mice have similar values to F1 (CBAxC57) (p > 0.73, Wilcoxon rank sum). C57BL/6 mice and F1 (CBAxC57) mice had similar RFS values in L4 neurons (p > 0.24, Wilcoxon rank sum) (Fig. 2G, right). However, Emx-TetO-GC6 mice and Thy1-GC6 mice have different RFS distributions in L4. Emx-TetO-GC6 mice have significantly larger RFS values than F1 (CBAxC57) (p < 3.9 × 10^–5^, Wilcoxon rank sum), yet Thy1-GC6 mice have significantly lower RFS values than F1 (CBAxC57) (p < 0.05, Wilcoxon rank sum). To investigate further, we split up RFS by sound level by summing the normalized receptive field across frequencies, rather than across both frequencies and sound levels (Fig. 2H). This gives a SPL-dependent profile of significant responses and their magnitude with respect to the rest of the RF. We find that in L2/3, C57BL/6 mice have a significantly higher RFS profile mean except at 20 dB where F1 (CBAxC57) is higher (Fig. 2H, left; Supplementary Table S1). A similar pattern was observed in L4 (Fig. 2H, right; Supplementary Table S1). From these results we observe that overall C57BL/6 mice, particularly Emx-TetO-GC6, had a much more drastic decrease in significant responses at quieter sound levels than in F1 (CBAxC57) mice. Since C57BL/6 L2/3 populations have narrower bandwidth (Fig. 2E, left), the higher RFS and RFS profile at high SPL in mice based on the Emx1-Cre may indicate that C57BL/6 mice have a larger response to a select few stimuli in their receptive field consistent with reduced inhibition^51^ and overall higher excitability of neurons labelled in the Emx1-Cre mouse line^53–54^ than in the neurons labelled in the Thy1-GC6 mice^55^. We conclude that F1 (CBAxC57) mice retain better hearing sensitivity to quieter sounds in old age.

Together, these analyses show that mid-frequency regions of A1 of C57BL/6 mice contain frequency selective neurons with a frequency preference that is shifted towards lower frequencies than that of F1 (CBAxC57) mice. However, the frequency selectivity and responsiveness of individual neurons from C57BL/6 mice is slightly higher than that of cells in F1 (CBAxC57) mice particularly in L2/3.

### F1 (CBAxC57) mice have a higher local heterogeneity of frequency preference than C57BL/6 mice

**Figure 3:**
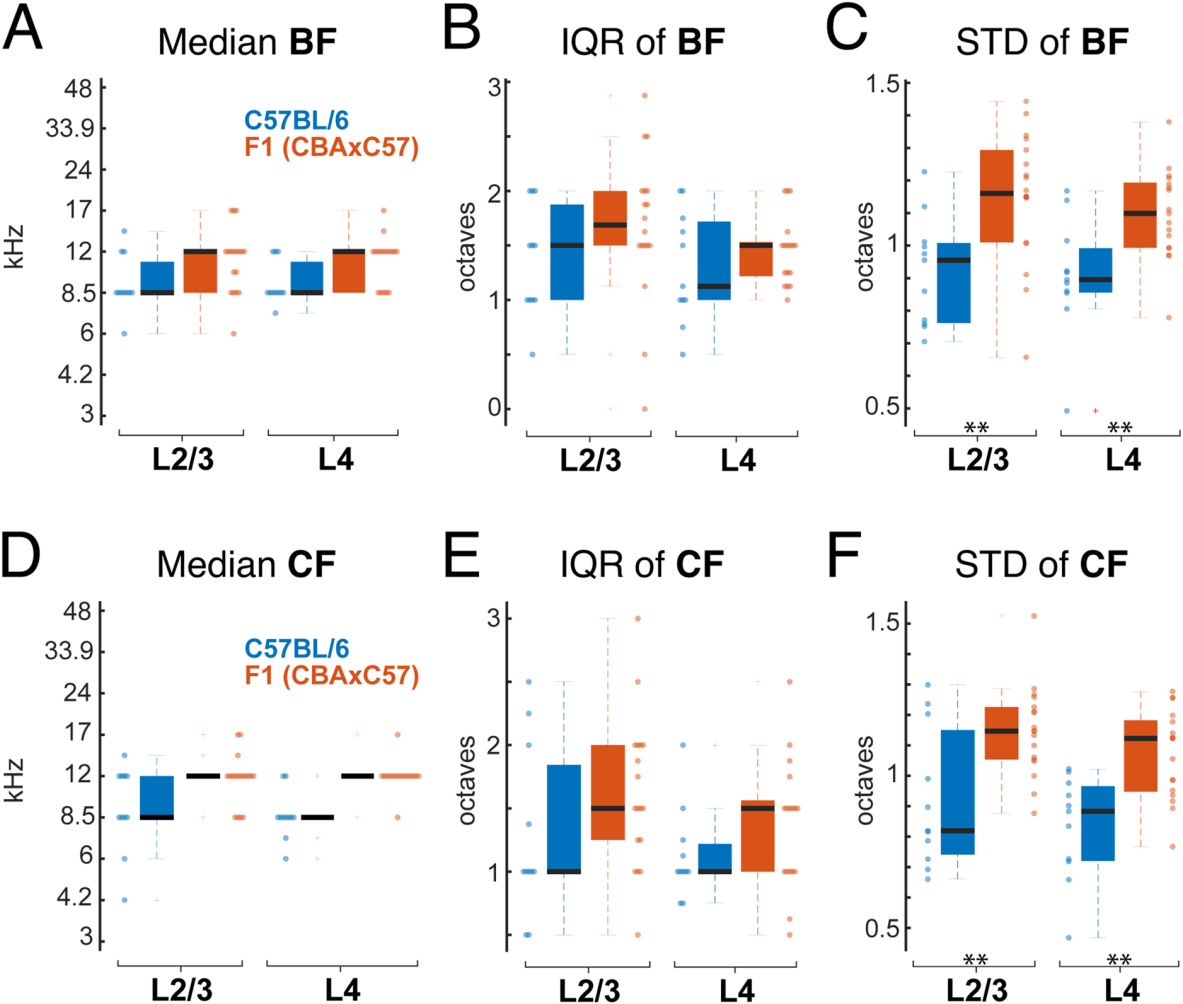
Tuning heterogeneity is higher in F1 (CBAxC57) mice. **(A)** Median BF for each FOV in L2/3 (left) and L4 (right). Each dot represents one FOV. Blue indicates C57BL/6 mice and orange indicates F1 (CBAxC57) mice. **(B)** IQR of BF in each FOV for L2/3 (left) and L4 (right). **(C)** STD of BF in each FOV for L2/3 (left) and L4 (right) populations. ** Indicates significant difference at p < 0.01 (Wilcoxon rank sum test or two-sample t-test depending on normality). **(D–F)** Conventions as in **(A–C)** but for CF. Statistical test details are included in Supplementary Table S2.

While on large scales A1 in rodents shows a tonotopic organization, nearby neurons can show different frequency preference^1–6,19–22^. We thus investigated if this local heterogeneity varied between mouse strains. We computed median, standard deviation (STD), and interquartile range (IQR) of the best frequency (BF) distribution for each FOV, where BF is the frequency that produced the highest response in each neuron regardless of sound pressure level. While IQR has been frequently used to assess auditory cortex (ACX) tuning heterogeneity, we further include the STD in order to include every observed tuned neuron in the field of view (i.e. even outliers). Consistent with the higher prevalence of high-frequency neurons in F1 (CBAxC57) mice (Fig. 2), the median BF of C57BL/6 FOVs in both L2/3 and L4 was 8.5 kHz while F1 (CBAxC57) mice has a median BF of 12 kHz in both layers (Fig. 3A). The IQR_BF_ in each FOV was slightly higher in F1 (CBAxC57) mice than C57BL/6 mice in both L2/3 and L4 (Fig. 3B). Despite the lack of significant difference of IQR_BF_ between strains, we observed significantly higher STD_BF_ of F1 (CBAxC57) mice in both L2/3 and L4 (Fig. 3C) (p_L2/3_ < 0.005, p_L4_ < 0.004, Wilcoxon rank sum and two-sample t-test respectively), indicating a strong presence of neurons tuned to vastly different frequencies than the rest of the FOV (i.e. outliers) in F1 (CBAxC57) mice.

While CF and BF are generally well correlated, we demonstrated (Fig. 2D) that they often differ due to FRA nonlinearities. We thus also quantified the median, IQR, and STD of CFs from each FOV. We found precisely the same trends between mouse strains as observed with BF. Specifically, we find that median CF was generally the same in both layers of C57BL/6 mice (8.5 kHz) and across both layers of F1 (CBAxC57) mice (12 kHz) (Fig. 3D). IQR_CF_ was higher in F1 (CBAxC57) mice than in C57BL/6 mice in both L2/3 and L4 (Fig. 3E). STD_CF_ was significantly higher in F1 (CBAxC57) mice than in C57BL/6 mice in both L2/3 and L4 (Fig. 3F) (p_L2/3_ < 0.004, p_L4_ < 10^–3^, two-sample t-test). These results reinforce our finding that frequency tuning heterogeneity of neuronal populations is present in both strains of mice, yet larger in F1 (CBAxC57) mice. The larger spread of preferred frequencies in F1 (CBAxC57) mice compared to C57BL/6 mice is likely due to the deficit of high frequency tuned neurons observed in neuronal populations of C57BL/6 mice.

### Increased tuning heterogeneity in F1 (CBAxC57) mice is present at multiple length scales

**Figure 4:**
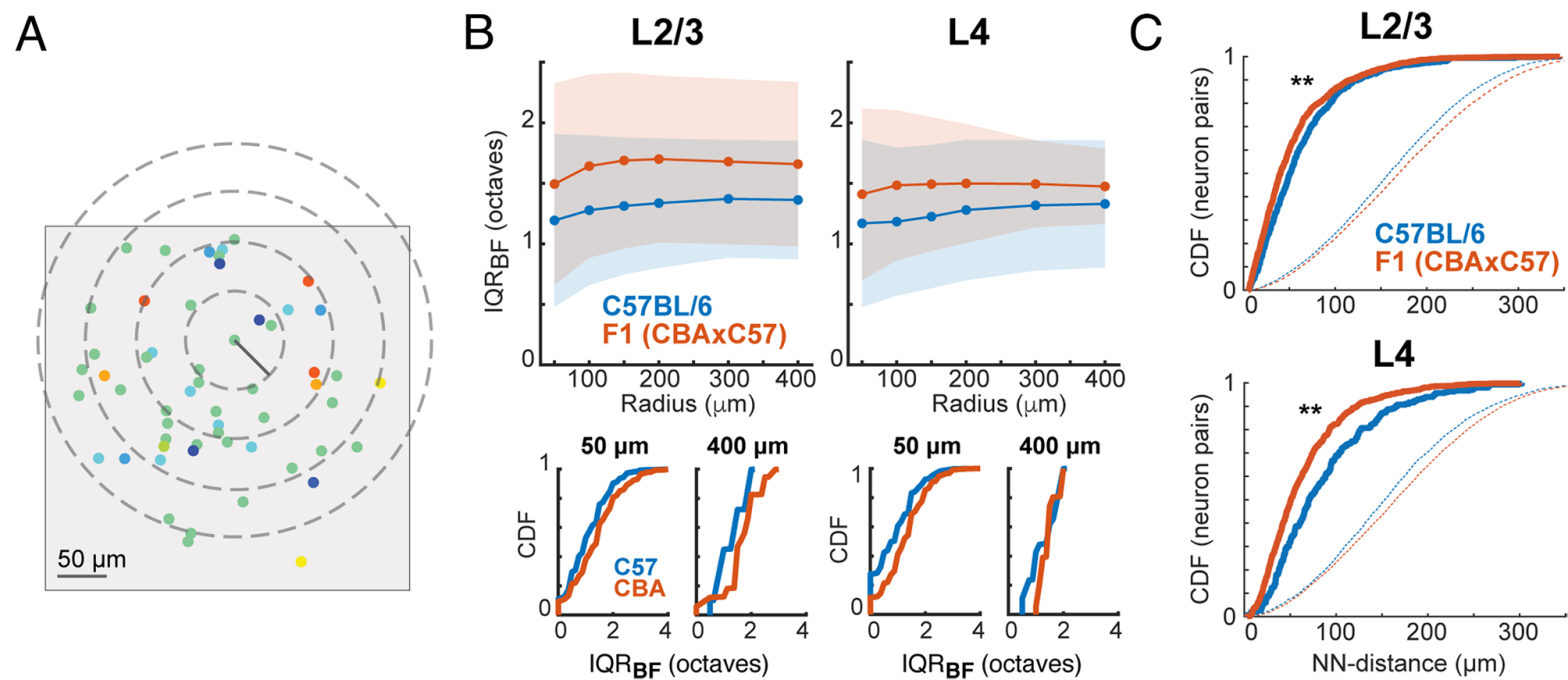
F1 (CBAxC57) mice have more tuning heterogeneity across length scales.** (A)** Schematic illustrating concentric circles (“neighborhoods”) of varying radii around a single neuron. Gray box indicates FOV. 300 µm and 400 µm circle not shown. **(B)** Top, Mean IQR_BF_ for each mouse strain at each length scale. Shading represents STD. Bottom, Cumulative distribution functions of IQR_BF_ for each mouse strain at short (50 µm) and long (400 µm) length scales. **(C)** Cumulative distribution functions of same-tuned nearest-neighbor distances for neuron pairs in each mouse strain. Median and IQR for each distribution: C57_L2/3_ = 51 (54), F1_L2/3_ = 40 (47), p < 4 × 10^–7^ (Wilcoxon rank sum); C57_L4_ = 70 (76), F1_L4_ = 51 (55) p < 1 × 10^–11^ (Wilcoxon rank sum). Dashed lines indicate randomly sampled pairwise distances from the neuronal populations.

We next investigated whether the differences in tuning heterogeneity between strains is restricted to small length scales or is maintained over larger populations of neurons. We calculated IQR_BF_ and IQR_CF_ at several different length scales, where the IQR is computed in a neighborhood surrounding each individual neuron. One neuron was picked as the central neuron and then IQR was computed from BFs of neurons within a radius ranging from 50 to 400 µm around that neuron (Fig. 4A). This process was repeated for each neuron in the FOV, using each one as the center of the local neighborhood. We found that F1 (CBAxC57) mice maintain a higher extent of frequency tuning heterogeneity across all length scales up to the size of our FOVs than C57BL/6 mice (Fig. 4B). Furthermore, we observed slightly lower IQR_BF_ values at smaller length scales, particularly in L2/3, indicating a small-scale localization of similarly tuned neurons.

We further analyzed the spatial heterogeneity of tuning preference by performing analysis outlined in a previous study^22^. We first computed the nearest-neighbor distance for each neuron in a FOV by finding the minimum distance to another neuron with the same tuning (Fig. 4C; solid lines). We compared these distributions to random distributions where an equal number of pairwise distances were randomly sampled from the possible pairwise distances between tuned neurons (Fig. 4C; dashed lines). We found that both F1 (CBAxC57) and C57BL/6 mice had nearest neighbor distances significantly smaller than random. F1 (CBAxC57) mice had smaller nearest-neighbor distances than C57BL/6 mice which serves as further support that despite the increased heterogeneity in F1 (CBAxC57) FOVs, the similarly tuned cells are more densely localized. Together these data show that the spatial heterogeneity in frequency preference does not depend on mouse strain and thus is a feature of rodent auditory cortex.

### Signal correlations are higher in C57BL/6 mice

**Figure 5:**
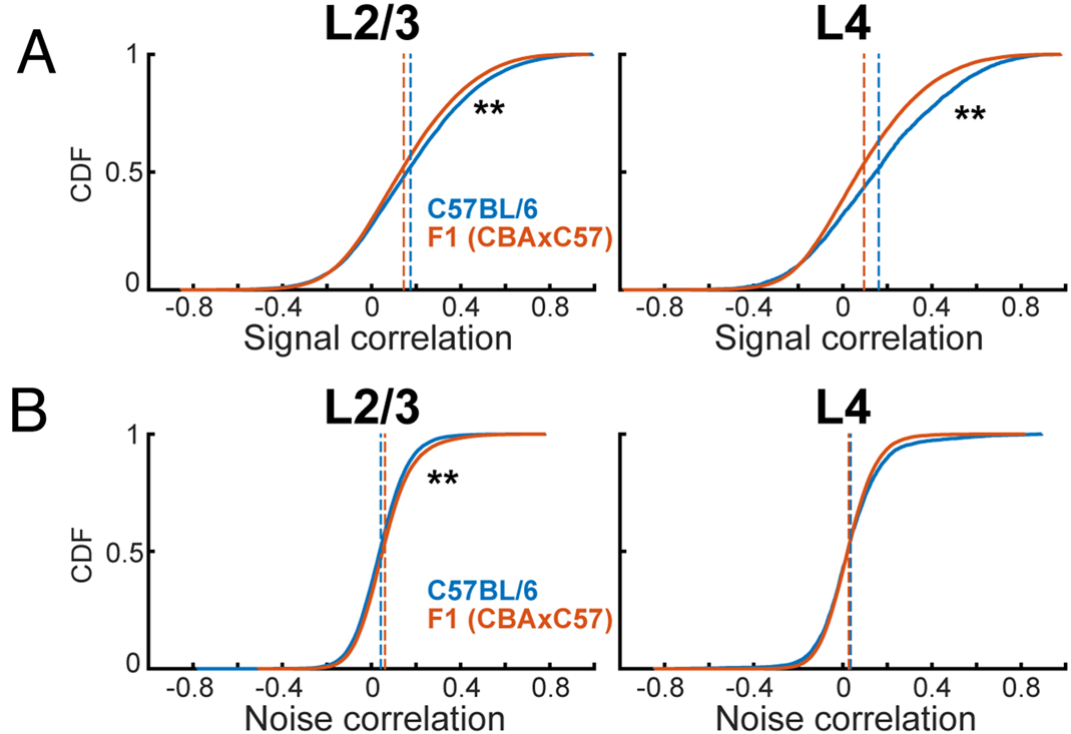
Signal and noise correlations across strains. **(A)** Cumulative distribution functions of signal correlations in both strains in L2/3 (left) and L4 (right). Dashed lines indicate distribution mean. ** Indicates significant difference at p < 0.01 (Wilcoxon rank sum test). **(B)** Conventions as in **(A)**, but for noise correlations. Statistical test details are included in Supplementary Table S3.

We next investigated if the slight differences we observed in receptive fields of each mouse strain were reflected in measures of functional connectivity. We calculated signal correlations which infer similar feedforward input to neuronal pairs by looking at the covariance between receptive fields of each pair. We observed non-normal distributions with a bias towards positive values for signal correlations. We found that signal correlations (SC) are higher in C57BL/6 mice in both L2/3 and L4 neuronal populations (Fig. 5A) (L2/3 mean: SC_C57_ = 0.17, SC_CBA_ = 0.14; L4 mean: SC_C57_ = 0.16, SC_CBA_ = 0.10). This indicates that overall, neuronal pairs in C57BL/6 mice have more similar inputs than in F1 (CBAxC57) mice. Next, we calculated noise correlations (NC) which infer direct connections between neurons or shared sources of activity perturbations. NCs centered closer to zero than SCs with a slight bias towards positive values. NCs in F1 (CBAxC57) mice trended slightly higher in L2/3 than in C57BL/6 mice (Fig. 5B) (L2/3 mean: NC_C57_ = 0.04, NC_F1_ = 0.06; L4 mean: NC_C57_ = 0.04, NC_F1_ = 0.03).

**Figure 6:**
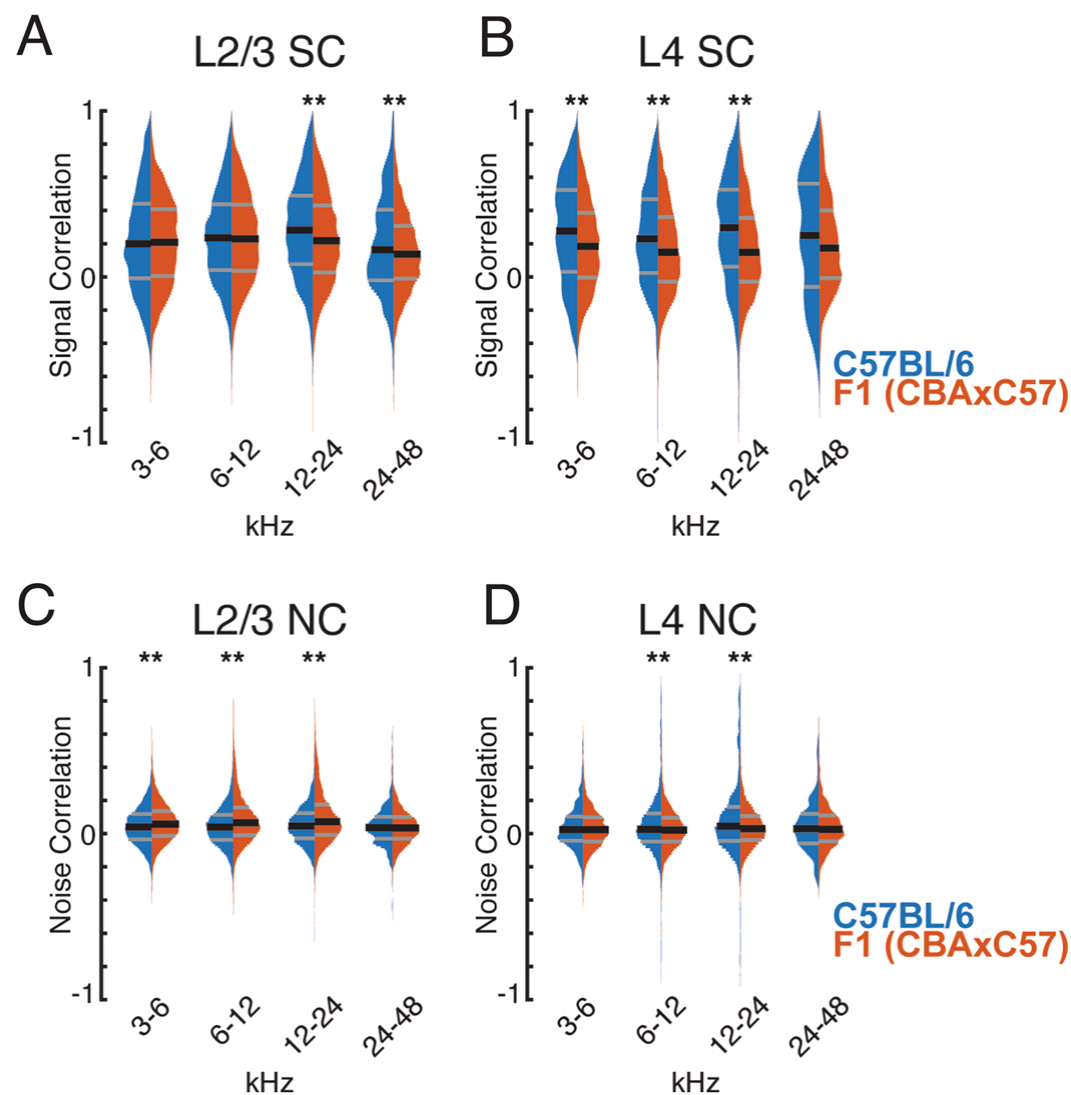
Signal and noise correlations of similarly tuned neurons. **(A)** Signal correlation distributions of similarly tuned neurons in L2/3 of both strains. ** Indicates significant difference at p < 0.01 (Wilcoxon rank sum test). **(B)** Conventions as in **(A)**, but for L4. **(C)** Noise correlation distributions of similarly tuned neurons in L2/3 of both strains. ** Indicates significant difference at p < 0.01 (Wilcoxon rank sum test). **(D)** Conventions as in **(C)**, but for L4. Statistical test details are included in Supplementary Table S4.

Since C57BL/6 mice have a decreased frequency representation of high frequencies we next investigated if changes in pairwise correlations were similar for cell pairs across the hearing range. We thus separately calculated pairwise correlations between cells with frequency preference in different octave bands. SCs in L2/3 were similar in 3–6 KHz and 6–12 kHz band between genotypes but were higher in C57BL/6 mice in 12–24 and 24–48 kHz (Fig. 6A). In L4, consistent with our overall observations, SCs were higher in most frequency bands, except for the 24–48 kHz band were only a trend was observed (Fig. 6B). These results show that C57BL/6 mice have higher SCs than F1 (CBAxC57) mice across most of the hearing range. NCs were larger for frequency bands < 24 KHz in CBA mice than in C57BL/6 mice (Fig. 6C), but NCs were largely similar in all frequency across the hearing range in L4 (Fig. 6D). Together, these results indicate that, while overall pairwise correlations between F1 (CBAxC57) and C57BL/6 are similar, small differences exist and these differences are present in the middle frequency ranges which do not show reduced representation in C57BL/6. These results suggest that there are subtle intrinsic differences in A1 processing between mouse strains.

**Figure 7:**
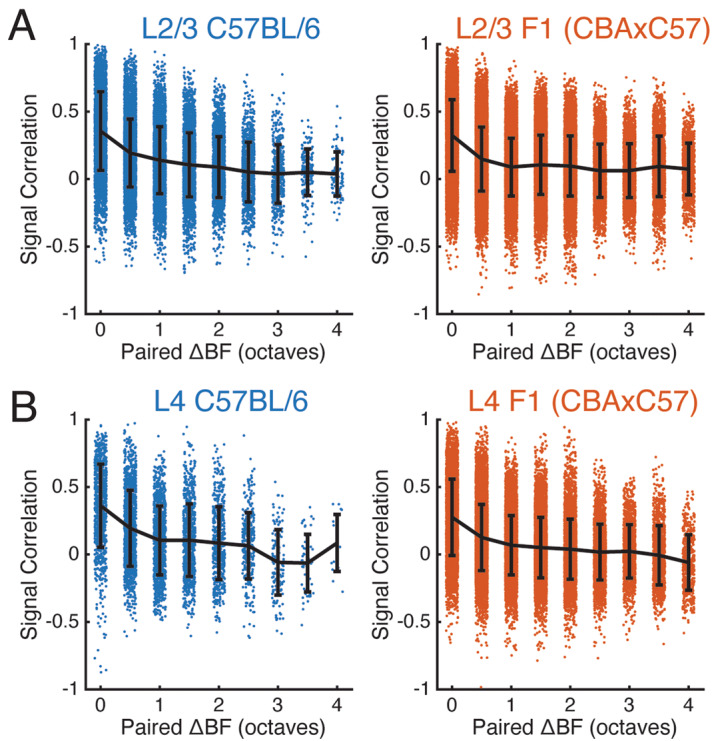
Signal correlations decrease as tuning preference deviates. **(A)** Signal correlation distributions of neuron pairs with respect to their difference in preferred frequency in L2/3 of both strains. **(B)** Conventions as in **(A)**, but for L4.

Given the observed tuning diversity in ACX neuronal populations, we next investigated how SCs differ depending on the BF relationships of the cell pair. We quantified the SCs of cell pairs as a function of difference in preferred frequency. We found that in L2/3 populations of both strains SCs drop off as BFs differ between neurons (Fig. 7A), even if the difference is as small as 0.5 octaves, and then remain relatively constant with respect to ΔBF. In L4 populations of both strains, SCs again drop off at a ΔBF of 0.5 octaves (Fig. 7B) and then remain relatively constant at differences greater than 1 octave. In addition to the slight shifts in means, both L2/3 and L4 populations of both strains have a wide distribution of SCs at all ranges of ΔBF. These results indicate that despite differences in receptive fields and tuning diversity of both mouse strains, pairwise activity relationships in activity seems largely unchanged.

## Discussion

Using in vivo 2-photon imaging we show that A1 in both the C57BL/6 and F1 (CBAxC57) strains of mice contains neurons largely similarly tuned and responsive for sound frequencies but that F1 (CBAxC57) mice contain more neurons selective for high frequencies. Moreover, we show that the spatial representation of sound frequency representation in A1 is heterogeneous in both strains of mice. Thus, the observed heterogeneity in prior studies using C57BL/6 mice is not due to changes in the cochlea and subsequent central plasticity in these mice and indicates that heterogeneous representation of sound preference is a feature of rodent auditory cortex.

C57BL/6 mice show progressive degeneration of the basal turn of the cochlea that becomes evident on a histological level by 3 months^30^. In addition, auditory brainstem response (ABR) differences can be observed at ~ 10 weeks^51,56^. However, both measures are relatively coarse, thus it is likely that subtle functional deficits are present at even earlier ages. The mice in our study were within this range consistent with their relative normal hearing. The main deficit we observed was a relative paucity of high-frequency (> 32 kHz) responding cells in C57BL/6 mice consistent with an emerging degeneration of peripheral high-frequency hearing and the changes on the ABR level^51,56^.

Functional and molecular differences between strains are present on the brainstem level at even younger ages but some of these could reflect strain differences unrelated to hearing loss. For example cells in the VNTB fire at lower rates in C57BL/6 mice than F1 (CBAxC57) mice at young ages^57^ and deficits in the efferent feedback precede overt hearing deficits and could be observed by 8 weeks^58,59^. By 6 months of age C57BL/6 mice also show a loss of parvalbumin immunoreactivity in A1 and AAF suggesting a loss or hypofunction of fast spiking (PV) inhibitory interneurons^51,60^. However, C57BL/6 mice show a higher number of PV cells than F1 (CBAxC57) mice at young ages indicating baseline strain differences in cortical circuits^60^. A loss of inhibition with age would be expected to result in changes in the functional responses, such as a broadening of FRAs. While we did observe a bandwidth difference between strains, our data showed higher frequency selectivity in C57BL/6 mice than F1 (CBAxC57) mice consistent with the higher baseline number of PV cells in these mice^60^. Additionally, any decrease in PV immunoreactivity in C57BL/6 mice might have not impaired the function of PV cells, or decreased PV cell function in C57BL/6 mice might have been compensated for by other classes of interneurons.

We observed both onset and offset responses consistent with prior reports^6^. Possibly due to our usage of shorter tone stimuli or SAM tones we observed a lower fraction of offset responsive neurons than our prior study^6^. Furthermore, here we used a stricter criterion for off-responsive neurons such that on-responsive neurons were disqualified from also being off-responsive neurons. Nevertheless, we observed an altered ratio of onset to offset neurons with C57BL/6 mice having fewer offset neurons that F1 (CBAxC57) mice. While onset and offset responsiveness is determined by distinct thalamocortical circuits, inhibitory circuits are thought to play a role in enhancing offset responses^6^. Thus, our observation here could indicate hypofunction of inhibition. Given that we do not observe broadening of receptive fields in C57BL/6 mice as compared to F1 (CBAxC57) mice, temporal effects of inhibitory hypofunction reflected in offset responses might be a more sensitive indicator of inhibitory hypofunction than pure tone bandwidth.

Thus, consistent with prior anatomical and ABR data our single cell results suggest that at ~ 10 weeks of age there is no large effect of hearing loss on A1 of C57BL/6 mice besides the paucity of high frequency neurons in mid-frequency regions. However, it is possible that neuroplastic changes have already been taking place to adjust the tuning of these former high-frequency neurons, compensating for peripheral changes.

Our results show that a local diversity of frequency tuning is present in mice of both strains. This confirms observations using in vivo 2-photon Ca^2+^-imaging under various conditions in multiple labs. Initial studies using synthetic dyes in anesthetized animals described a local heterogeneous frequency preference^1,2^ in L2/3. These results in L2/3 have been confirmed using the sensitive synthetic dye Cal-520^3,4,22^ in anesthetized and awake mice and rats, with the ultrasensitive genetically encoded calcium indicator (GECIs) GCaMP6s in awake mice^5,6^, as well as by in vivo patch clamp studies^61^. In addition to primary auditory cortex, L2/3 of ACX areas such as the anterior auditory field and secondary ACX were also shown to contain fractured tonotopic organization^6^. Interestingly, using low sensitivity indicators (GCaMP3) revealed a high degree of local similarity^18^ suggesting that strongly responding neurons in a local field might be very similarly tuned possibly due to subsampling certain classes of L2/3 neurons^5^. Investigation of the tuning of MGB afferents in A1 showed that the local frequency distribution of MGB terminals was very heterogeneous^6,62^. Thus at least in mouse a large degree of local tuning diversity is present on multiple levels in the auditory cortex. Further studies using the synthetic dye Fluo-4 revealed that L4 showed less local tuning diversity than L2/3^19^ consistent with electrophysiological results^21,63^. The increased diversity in L2/3 over L4 suggest that processing complexity might increase in superficial layers consistent with recent findings in humans^64,65^. Interestingly, studies in marmoset find local tuning homogeneity in marmoset A1 which indicates species differences in local A1 organization^22^.

In general, the degree of heterogeneity in the local frequency organization varies between prior rodent studies. In particular, recent imaging using the synthetic dye Cal-520 found no difference between layers^4^. While these differences between studies might be due to differences in the experimental conditions, cell inclusion criteria, range of tonal stimuli and sound levels used, other factors might play a role. We speculate that the differences in local frequency preference might reflect plastic changes due to different rearing environments. Exposure to sounds in development can alter the functional organization of A1^66–68^. Since rodent housing can be loud and might vary considerably between institutions^69^, it is possible that differences seen between studies reflect sensory adaptations to the local environment or low levels of hearing loss induced by personal activity in the local environment. Support for this hypothesis comes from studies in other systems. Local heterogeneity of tuning in L2/3 is a feature of not only rodent A1 but also of primary visual and somatosensory cortices^70–72^. For example, S1 local L4 neurons show very similar tuning, yet L2/3 neurons are more diverse^71^. This difference between layers was dependent on mice living in impoverished versus enriched environments suggesting that experience is shaping the differential representation of stimulus properties in L4 and L2/3. Our study investigated A1 in two strains of mice raised in the same sound environment. Our colony room is equipped with silent door latches and sound dampening panels reducing the occurrence of loud transient and steady state background sounds^69^. Our results in mice of two strains using genetically encoded Ca^2+^ indicators show similar functional organization of A1 as our prior studies using synthetic dyes^19^. Thus, the local tuning diversity especially in L2/3 is a common property of rodent auditory cortex.

Our measurements of signal correlations were generally less positive than previously reported results^19^, potentially due to lower frame rates and spatial resolution in prior work. In contrast to these prior studies we calculated the covariance of responses across multiple sound levels rather than one sound level in order to more fully capture the interactions between neurons. Receptive fields of ACX neurons are highly nonlinear with respect to sound level, and as a result our inputs to the covariance measure are more variable than when using one sound level.

In summary, our results suggest that A1 of C57BL/6 in adult mice is very similarly responsive and similarly organized to A1 of F1 (CBAxC57) mice. Thus, while aspects of cellular tuning differ between strains, the differences in cortical processing at this age is modest at best and largely restricted to a paucity of high frequency neurons.

## Supplementary information

Supplementary file1 (PDF 193 kb)

## References

[1] Bandyopadhyay S, Shamma SA, Kanold PO (2010). Dichotomy of functional organization in the mouse auditory cortex. Nat Neurosci.

[2] Rothschild G, Nelken I, Mizrahi A (2010). Functional organization and population dynamics in the mouse primary auditory cortex. Nat. Neurosci..

[3] Li J (2017). Functional imaging of neuronal activity of auditory cortex by using Cal-520 in anesthetized and awake mice. Biomed. Opt. Express.

[4] Tischbirek CH (2019). In vivo functional mapping of a cortical column at single-neuron resolution. Cell Rep..

[5] Meng X, Winkowski DE, Kao JP, Kanold PO (2017). Sublaminar subdivision of mouse auditory cortex layer 2/3 based on functional translaminar connections. J. Neurosci..

[6] Liu J (2019). Parallel processing of sound dynamics across mouse auditory cortex via spatially patterned thalamic inputs and distinct areal intracortical circuits. Cell Rep..

[7] Merzenich MM, Knight PL, Roth GL (1975). Representation of cochlea within primary auditory cortex in the cat. J. Neurophysiol..

[8] Reale RA, Imig TJ (1980). Tonotopic organization in auditory cortex of the cat. J. Comp. Neurol..

[9] Stiebler I, Neulist R, Fichtel I, Ehret G (1997). The auditory cortex of the house mouse: Left-right differences, tonotopic organization and quantitative analysis of frequency representation. J. Comp. Physiol. A.

[10] Baba H (2016). Auditory cortical field coding long-lasting tonal offsets in mice. Sci. Rep..

[11] Bizley JK, Nodal FR, Nelken I, King AJ (2005). Functional organization of ferret auditory cortex. Cereb. Cortex.

[12] Nelken I (2004). Large-scale organization of ferret auditory cortex revealed using continuous acquisition of intrinsic optical signals. J. Neurophysiol..

[13] van Dijk P, Langers DR (2013). Mapping tonotopy in human auditory cortex. Adv. Exp. Med. Biol..

[14] Woods DL (2010). Functional properties of human auditory cortical fields. Front. Syst. Neurosci..

[15] Wessinger CM, Buonocore MH, Kussmaul CL, Mangun GR (1997). Tonotopy in human auditory cortex examined with functional magnetic resonance imaging. Hum. Brain Mapp..

[16] Striem-Amit E, Hertz U, Amedi A (2011). Extensive cochleotopic mapping of human auditory cortical fields obtained with phase-encoding FMRI. PLoS ONE.

[17] Moerel M, De Martino F, Formisano E (2014). An anatomical and functional topography of human auditory cortical areas. Front. Neurosci..

[18] Issa JB (2014). Multiscale optical Ca2+ imaging of tonal organization in mouse auditory cortex. Neuron.

[19] Winkowski DE, Kanold PO (2013). Laminar transformation of frequency organization in auditory cortex. J. Neurosci..

[20] Panniello M, King AJ, Dahmen JC, Walker KMM (2018). Local and global spatial organization of interaural level difference and frequency preferences in auditory cortex. Cereb. Cortex.

[21] Kanold PO, Nelken I, Polley DB (2014). Local versus global scales of organization in auditory cortex. Trends Neurosci..

[22] Zeng HH (2019). Local homogeneity of tonotopic organization in the primary auditory cortex of marmosets. Proc. Natl. Acad. Sci. USA.

[23] Mock BE, Vijayakumar S, Pierce J, Jones TA, Jones SM (2016). Differential effects of Cdh 23(753A) on auditory and vestibular functional aging in C57BL/6J mice. Neurobiol. Aging.

[24] Miyasaka Y (2013). Compound heterozygosity of the functionally null Cdh23(v-ngt) and hypomorphic Cdh23(ahl) alleles leads to early-onset progressive hearing loss in mice. Exp. Anim..

[25] Kane KL (2012). Genetic background effects on age-related hearing loss associated with Cdh23 variants in mice. Hear Res..

[26] Johnson KR (2010). Separate and combined effects of Sod1 and Cdh23 mutations on age-related hearing loss and cochlear pathology in C57BL/6J mice. Hear Res..

[27] Zheng QY, Ding D, Yu H, Salvi RJ, Johnson KR (2009). A locus on distal chromosome 10 (ahl4) affecting age-related hearing loss in A/J mice. Neurobiol. Aging.

[28] Di Palma F, Pellegrino R, Noben-Trauth K (2001). Genomic structure, alternative splice forms and normal and mutant alleles of cadherin 23 (Cdh23). Gene.

[29] Zheng QY (2005). Digenic inheritance of deafness caused by mutations in genes encoding cadherin 23 and protocadherin 15 in mice and humans. Hum. Mol. Genet..

[30] Park SN (2010). Comparison of cochlear morphology and apoptosis in mouse models of presbycusis. Clin. Exp. Otorhinolaryngol..

[31] Hunter KP, Willott JF (1987). Aging and the auditory brainstem response in mice with severe or minimal presbycusis. Hear Res..

[32] Willott JF, Carlson S, Chen H (1994). Prepulse inhibition of the startle response in mice: Relationship to hearing loss and auditory system plasticity. Behav. Neurosci..

[33] Li HS, Borg E (1991). Age-related loss of auditory sensitivity in two mouse genotypes. Acta Otolaryngol..

[34] Ouagazzal AM, Reiss D, Romand R (2006). Effects of age-related hearing loss on startle reflex and prepulse inhibition in mice on pure and mixed C57BL and 129 genetic background. Behav. Brain Res..

[35] Parham K, Willott JF (1988). Acoustic startle response in young and aging C57BL/6J and CBA/J mice. Behav. Neurosci..

[36] Frisina RD (2011). F1 (CBA× C57) mice show superior hearing in old age relative to their parental strains: Hybrid vigor or a new animal model for “golden ears”?. Neurobiol. Aging.

[37] Kuchibhotla KV (2017). Parallel processing by cortical inhibition enables context-dependent behavior. Nat. Neurosci..

[38] Resnik, J. & Polley, D. B. Fast-spiking GABA circuit dynamics in the auditory cortex predict recovery of sensory processing following peripheral nerve damage. *Elife* **6**10.7554/eLife.21452 (2017). 10.7554/eLife.21452 PMC5378474 28323619

[39] Guo W, Clause AR, Barth-Maron A, Polley DB (2017). A corticothalamic circuit for dynamic switching between feature detection and discrimination. Neuron.

[40] Carcea I, Insanally MN, Froemke RC (2017). Dynamics of auditory cortical activity during behavioural engagement and auditory perception. Nat. Commun..

[41] Francis NA (2018). Small networks encode decision-making in primary auditory cortex. Neuron.

[42] Zheng QY, Johnson KR, Erway LC (1999). Assessment of hearing in 80 inbred strains of mice by ABR threshold analyses. Hear Res..

[43] Bowen, Z., Winkowski, D. E., Seshadri, S., Plenz, D. & Kanold, P. O. Neuronal Avalanches in input and associative layers of auditory cortex. *Front. Syst. Neurosci.* **13.**10.3389/fnsys.2019.00045 (2019). 10.3389/fnsys.2019.00045 PMC6737089 31551721

[44] Guo ZV (2014). Procedures for behavioral experiments in head-fixed mice. PLoS ONE.

[45] Goldey GJ (2014). Removable cranial windows for long-term imaging in awake mice. Nat. Protoc..

[46] Schindelin J (2012). Fiji: An open-source platform for biological-image analysis. Nat. Methods.

[47] Thevenaz P, Ruttimann UE, Unser M (1998). A pyramid approach to subpixel registration based on intensity. IEEE Trans. Image Process..

[48] Guizar-Sicairos M, Thurman ST, Fienup JR (2008). Efficient subpixel image registration algorithms. Opt. Lett..

[49] Chen TW (2013). Ultrasensitive fluorescent proteins for imaging neuronal activity. Nature.

[50] Peron S, Chen T-W, Svoboda K (2015). Comprehensive imaging of cortical networks. Curr. Opin. Neurobiol..

[51] Martin del Campo HN, Measor KR, Razak KA (2012). Parvalbumin immunoreactivity in the auditory cortex of a mouse model of presbycusis. Hear Res..

[52] Gorski JA (2002). Cortical excitatory neurons and glia, but not GABAergic neurons, are produced in the Emx1-expressing lineage. J. Neurosci..

[53] Steinmetz, N. A. *et al.* Aberrant cortical activity in multiple GCaMP6-expressing transgenic mouse lines. *eneuro* (2017). 10.1523/ENEURO.0207-17.2017 PMC5604087 28932809

[54] Chan C-H (2001). Emx1 is a marker for pyramidal neurons of the cerebral cortex. Cereb. Cortex.

[55] Dana H (2014). Thy1-GCaMP6 transgenic mice for neuronal population imaging in vivo. PLoS ONE.

[56] Ison JR, Allen PD, O'Neill WE (2007). Age-related hearing loss in C57BL/6J mice has both frequency-specific and non-frequency-specific components that produce a hyperacusis-like exaggeration of the acoustic startle reflex. J. Assoc. Res. Otolaryngol..

[57] Sinclair JL, Barnes-Davies M, Kopp-Scheinpflug C, Forsythe ID (2017). Strain-specific differences in the development of neuronal excitability in the mouse ventral nucleus of the trapezoid body. Hear Res..

[58] Zhu X (2007). Auditory efferent feedback system deficits precede age-related hearing loss: Contralateral suppression of otoacoustic emissions in mice. J. Comp. Neurol..

[59] Frisina RD, Newman SR, Zhu X (2007). Auditory efferent activation in CBA mice exceeds that of C57s for varying levels of noise. J. Acoust. Soc. Am..

[60] Brewton DH, Kokash J, Jimenez O, Pena ER, Razak KA (2016). Age-related deterioration of perineuronal nets in the primary auditory cortex of mice. Front. Aging Neurosci..

[61] Maor I, Shalev A, Mizrahi A (2016). Distinct spatiotemporal response properties of excitatory versus inhibitory neurons in the mouse auditory cortex. Cereb. Cortex.

[62] Vasquez-Lopez, S. A. *et al.* Thalamic input to auditory cortex is locally heterogeneous but globally tonotopic. *eLife* **6** (2017). 10.7554/eLife.25141 PMC5614559 28891466

[63] Guo W (2012). Robustness of cortical topography across fields, laminae, anesthetic states, and neurophysiological signal types. J. Neurosci..

[64] Moerel M, De Martino F, Ugurbil K, Yacoub E, Formisano E (2019). Processing complexity increases in superficial layers of human primary auditory cortex. Sci. Rep..

[65] Moerel M, De Martino F, Ugurbil K, Formisano E, Yacoub E (2018). Evaluating the columnar stability of acoustic processing in the human auditory cortex. J. Neurosci..

[66] Chang EF, Merzenich MM (2003). Environmental noise retards auditory cortical development. Science.

[67] Zhang LI, Bao S, Merzenich MM (2002). Disruption of primary auditory cortex by synchronous auditory inputs during a critical period. Proc. Natl. Acad. Sci. USA.

[68] Zhang LI, Bao S, Merzenich MM (2001). Persistent and specific influences of early acoustic environments on primary auditory cortex. Nat. Neurosci..

[69] Lauer AM, May BJ, Hao ZJ, Watson J (2009). Analysis of environmental sound levels in modern rodent housing rooms. Lab. Anim. (NY).

[70] Bonin V, Histed MH, Yurgenson S, Reid RC (2011). Local diversity and fine-scale organization of receptive fields in mouse visual cortex. J. Neurosci..

[71] LeMessurier, A. M. *et al.* Enrichment drives emergence of functional columns and improves sensory coding in the whisker map in L2/3 of mouse S1. *eLife* **8** (2019). 10.7554/eLife.46321 PMC6697414 31418693

[72] Ohki K, Reid RC (2007). Specificity and randomness in the visual cortex. Curr. Opin. Neurobiol..

