# Supplementary material for: Functional organization of mouse primary auditory cortex in adult C57BL/6 and F1 (CBAxC57) mice
Source: Sci Rep. 2020 Jul 2;10:10905. doi: 10.1038/s41598-020-67819-4 (PMC7331716; doi:10.1038/s41598-020-67819-4)
Supplement: Supplementary file 1 — Supplementary file1 (PDF 193 kb) [file 41598_2020_67819_MOESM1_ESM.pdf]

Supplementary Information for:

Functional Organization of Mouse Primary Auditory Cortex in adult C57BL/6 and F1 (CBAxC57) mice

Zac Bowen, Daniel E. Winkowski, Patrick O. Kanold

Department of Biology, University of Maryland, College Park, MD 20742

Supplementary Figures

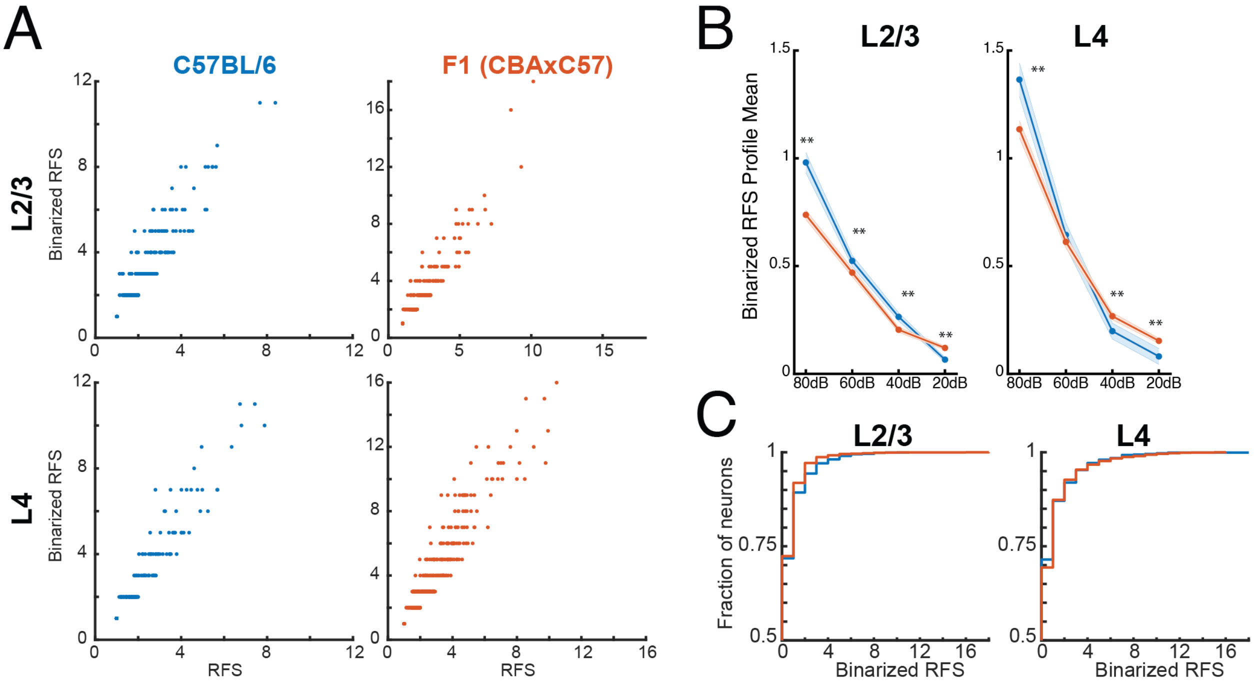

Supplementary Figure S1. Binarized receptive field sum results.
A) Receptive field sum plotted against binarized RFS. Each point represents one neuron. **B)** Receptive field sum split up by sound level. Sum is taken across rows in of the receptive field and then averaged across all neurons. ** indicates significance between C57BL/6 and F1 (CBAxC57) at p<0.01 (Wilcoxon rank sum test). **C)** Cumulative distribution functions of receptive field sum values in C57BL/6 (blue) and F1 (CBAxC57) (orange) mouse strains in both L2/3 (left) and L4 (right).

Supplementary Tables

	L2/3				L4			
	80dB	60dB	40dB	20dB	80dB	60dB	40dB	20dB
C57BL/6	0.028	0.034	1.1×10^{-3}	3.0×10^{-4}	1.3×10^{-3}	0.40	5.5×10^{-3}	4.2×10^{-5}
Emx-TetO-GC6	1.3×10^{-5}	0.048	0.30	1.7×10^{-3}	1.8×10^{-6}	0.10	0.17	1.0×10^{-3}
Thy1-GC6	0.07	0.23	7.1×10^{-6}	0.034	0.73	0.80	7.6×10^{-3}	8.8×10^{-3}

Supplementary Table S1. Statistical comparison to F1 (CBAxC57) RFS Profile.
P-values returned from Wilcoxon rank sum test between the listed quantity and the F1 (CBAxC57) values for Figure 2H.

	L2/3			L4		
	C57BL/6 (N=11)	F1 (CBAxC57) (N=18)	p-val and test	C57BL/6 (N=11)	F1 (CBAxC57) (N=17)	p-val and test
**Median BF**	4.00 (0.75)	5.00 (1)	0.06 (rank sum)	4.00 (0.75)	5.00 (1)	0.02 (rank sum)
**IQR of BF**	1.50 (0.88)	1.69 (0.5)	0.14 (rank sum)	1.13 (0.72)	1.50 (0.31)	0.33 (rank sum)
**STD of BF**	0.96 (0.25)	1.16 (0.28)	5.0×10^{-3} (rank sum)	0.90 (0.14)	1.10 (0.2)	3.8×10^{-3} (t-test)
**Median CF**	4.00 (1)	5.00 (0)	0.07 (rank sum)	4.00 (0)	5.00 (0)	8.5×10^{-5} (rank sum)
**IQR of CF**	1.00 (0.84)	1.50 (0.75)	0.14 (rank sum)	1.00 (0.22)	1.50 (0.56)	0.19 (rank sum)
**STD of CF**	0.82 (0.41)	1.15 (0.17)	3.1×10^{-3} (t-test)	0.88 (0.25)	1.12 (0.23)	5.8×10^{-4} (t-test)

Supplementary Table S2. Distribution statistics for BF and CF measures.
Median value and IQR are displayed for each quantity in Figure 3. All values are in octaves. Reported p-values were calculated using Wilcoxon rank sum test or two-sample t-test depending on normality.

	L2/3			L4		
	C57BL/6 (N=20492)	F1 (CBAxC57) (N=67147)	p-val and test	C57BL/6 (N=5165)	F1 (CBAxC57) (N=38843)	p-val and test
**Sig. Corr.**	0.16 (0.37)	0.13 (0.34)	1.6×10^{-40} (rank sum)	0.15 (0.43)	0.07 (0.34)	8.2×10^{-55} (rank sum)
**Noise Corr.**	0.04 (0.14)	0.05 (0.15)	5.4×10^{-61} (rank sum)	0.02 (0.16)	0.02 (0.14)	0.19 (rank sum)

Supplementary Table S3. Distribution statistics for signal and noise correlations.
Median value and IQR are displayed for each distribution of correlations in Figure 5. Reported p-values were calculated using Wilcoxon rank sum test.

	L2/3 Signal Correlations				L4 Signal Correlations			
	3-6kHz	6-12kHz	12-24kHz	24-48kHz	3-6kHz	6-12kHz	12-24kHz	24-48kHz
C57BL/6	0.20 (0.45)	0.24 (0.40)	0.28 (0.41)	0.16 (0.43)	0.28 (0.49)	0.23 (0.44)	0.30 (0.46)	0.25 (0.62)
F1 (CBAxC57)	0.21 (0.40)	0.23 (0.40)	0.22 (0.40)	0.14 (0.32)	0.18 (0.39)	0.15 (0.39)	0.15 (0.38)	0.17 (0.41)
**p-val**	0.30	0.23	5.1×10^{-22}	3.5×10^{-3}	2.8×10^{-8}	9.3×10^{-22}	5.4×10^{-29}	0.12
	**L2/3 Noise Correlations**				**L4 Noise Correlations**			
	3-6kHz	6-12kHz	12-24kHz	24-48kHz	3-6kHz	6-12kHz	12-24kHz	24-48kHz
C57BL/6	0.04 (0.15)	0.04 (0.15)	0.05 (0.15)	0.04 (0.13)	0.02 (0.14)	0.03 (0.17)	0.04 (0.21)	0.03 (0.18)
F1 (CBAxC57)	0.06 (0.15)	0.07 (0.17)	0.07 (0.18)	0.03 (0.13)	0.02 (0.15)	0.02 (0.14)	0.03 (0.14)	0.03 (0.16)
**p-val**	8.8×10^{-10}	2.4×10^{-82}	1.9×10^{-35}	0.69	0.65	6.5×10^{-4}	2.6×10^{-6}	0.98

Supplementary Table S4. Distribution statistics for correlations in each BF band.
Median value and IQR are displayed for each distribution of correlations in Figure 6. Reported p-values were calculated using Wilcoxon rank sum test.
